# Porcine placental extract facilitates memory and learning in aged mice

**DOI:** 10.1002/fsn3.1156

**Published:** 2019-08-15

**Authors:** Akihiro Yamauchi, Takahiro Tone, Koji Sugimoto, Hong Seok Lim, Taiichi Kaku, Chihiro Tohda, Takayuki Shindo, Koji Tamada, Yoichi Mizukami, Eiichi Hirano

**Affiliations:** ^1^ Research Institute Japan Bio Products Co., Ltd. Kurume Japan; ^2^ Japan Bio Products Co., Ltd. Kurume Japan; ^3^ Division of Neuromedical Science, Department of Bioscience, Institute of Natural Medicine University of Toyama Toyama Japan; ^4^ Department of Cardiovascular Research Shinshu University Graduate School of Medicine Nagano Japan; ^5^ Department of Immunology, Graduate School of Medicine Yamaguchi University Yamaguchi Japan; ^6^ Institute of Gene Research Yamaguchi University Science Research Center Yamaguchi Japan

**Keywords:** aging, hippocampus, memory, porcine placental extract, RNA‐seq

## Abstract

Aging induces a decline in both memory and learning ability without predisposing an individual to diseases of the central nervous system, such as dementia. This decline can have a variety of adverse effects on daily life, and it can also gradually affect the individual and the people they are surrounded by. Since recent evidence indicated that placental extract has effects on brain function such as memory, we hypothesized that placental extract could ameliorate the age‐associated reduction in cognitive function in aging. Here, we investigated the effect of new modified porcine placental extract (SD‐F) on memory ability in aged mice at both the behavioral and molecular levels. Our results revealed that SD‐F significantly enhanced memory ability in the object recognition and object location tasks in a dose‐dependent manner in aged mice relative to controls. The numbers of Nissl‐positive cells in the hippocampal cornu ammonis 3 (CA3) and dentate gyrus (DG) regions were increased in SD‐F‐treated aged mice relative to controls. RNA‐seq analysis of the hippocampus of aged mice identified 542 differentially expressed genes, of which 216 were up‐regulated and 326 were down‐regulated in SD‐F‐treated mice relative to controls. Of the 216 up‐regulated genes, we identified four characteristic genes directly related to memory, including *early growth response protein 1* (*Egr1*), *growth arrest and DNA‐damage‐inducible, beta* (*Gadd45b*), *NGFI‐A binding protein 2* (*Nab2*), and *vascular endothelial growth factor a* (*Vegfa*). These results suggest that the efficacy of SD‐F involves upregulation of these genes.

## 1 INTRODUCTION

Aging commonly occurs in all people with several changes in body chemistry (St‐Onge, 2005), and it is complex and coherent. Aging is also a prime risk factor for various diseases and gives rise to a decline in cognitive function through the major forms of neurodegeneration, such as Alzheimer's disease (AD), for which aging is the most significant risk factor (Bertram & Tanzi, 2008). Age‐associated memory impairment is observed in rodents as well as in humans (Bishop, Lu, & Yankner, 2010; Verdaguer et al., 2012), and research and development has promoted the generation of drugs to ameliorate such impairments, although there has been no success yet. The reason for this may be that the mechanisms underlying memory impairment are complex, and a single molecular target would be insufficient to explain such impairments.

Placental extract can be simply obtained by hydrolysis of the placenta via both hydrochloric acid and enzymatic digestion, and it has been reported to have many biological activities in vitro and in vivo (Bak et al., 2018, 2019; Ito, Yamada, Matsumoto, & Imamura, 2018; Yamauchi et al., 2017; Yoshimoto et al., 2018). There are now several placental extract products available, and these have been used as drugs or in health care. Clinically, placental extract has been administered to improve liver function in patients with hepatitis or cirrhosis in Japan, South Korea, and Russia. They have also been used as health supplements in general health care. Interestingly, it has been reported that placental extract improves memory impairment in an AD mouse model (Kogure & Tohda, 2017) or in chronically stressed mice (Park et al., 2018; Takuma et al., 2012), indicating that placental extract may impact on brain function. However, no study has investigated the effect of placental extract on memory deficits associated with aging. Based on the above, we hypothesized that placental extract may ameliorate the age‐associated decrease in cognitive function.

In this study, we investigated the effect of a new modified porcine placental extract (SD‐F) on memory deficits in aged mice at the behavioral and molecular levels to clarify the mechanistic effects of SD‐F.

## 2 MATERIALS AND METHODS

### 2.1 Materials

The new modified porcine placental extract (JBP code# SD‐F), which is a main raw material for JBP placenta jelly pure (product code is 1312.1 JBP PlacentaJellyPURE), was produced by Japan Bio Products Co., Ltd. Briefly, the porcine placenta collected in Japan is treated with protease, filtration, and heat sterilization and then spray‐dried. A standard laboratory diet, AIN‐93G (Oriental Yeast), and powder of porcine placenta extract (Japan Bio Products Co., Ltd.) were used. SD‐F containing diet was prepared by mixing both AIN‐93G powder and SD‐F powder so that the mice ingested 500, 1,000, and 5,000 mg/day of SD‐F per kg of body weight.

### 2.2 Animals and treatments

**Figure 1:**
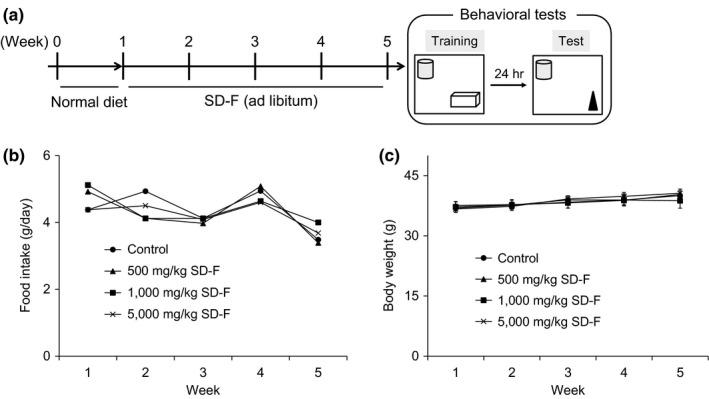
Experimental protocol and effect of SD‐F on food intake and body weight in aged mice. Schematic drawing of experimental protocol. After normal diet for one week, mice were divided into four groups (*n* = 9 per group), and then SD‐F (500, 1,000, or 5,000 mg/kg) or control (normal diet) was provided ad libitum for 4 weeks. Four weeks after ingestion, behavioral tests were carried out (a). Effect of SD‐F on food intake (b) and body weight (c). (closed circle) control group; (closed triangle) 500 mg/kg SD‐F‐treated group; (closed square) 1,000 mg/kg treated SD‐F group; (crosses) 5,000 mg/kg SD‐F‐treated group. There were no significant differences among the groups. Results represent means ± *SEM*

Mice were maintained under specific pathogen‐free conditions in an environmentally controlled clean room at the Division of Laboratory Animal Research, Department of Life Science, Research Center for Human and Environmental Sciences, Shinshu University. All animal handling procedures were performed in accordance with protocols approved by the Ethics Committee of the International Animal Care and Use Committee and NIH guidelines. Wild‐type C57BL/6J male mice, aged 19 months, were purchased from a supplier of experimental animals (CLEA Japan, Inc.). After receiving a normal diet and water ad libitum for 1 week, thirty‐six mice were randomly divided into 4 groups (*n* = 9/group): Group 1 mice were fed with a normal diet (control), and groups 2 to 4 were fed with an SD‐F containing diet (500, 1,000, and 5,000 mg/kg), respectively. The experimental protocol is shown in Figure 1a. The body weight and food intake of each mouse were measured weekly. Four weeks after the experiment began, behavioral analyses were performed and then mice were killed for further analyses.

### 2.3 Behavioral tests

#### 2.3.1 Object recognition test

Two identical objects were placed at a fixed distance within a square box. A mouse was then placed in the center of the box, and the number of times it contacted the two objects was recorded during a 10‐min period (training session). After a 24‐hr interval, the mice were placed back into the same box in which one of the objects used in the training session was replaced with a novel object. The mice were then allowed to explore freely for 10 min, and the number of times they contacted each object was recorded (test session). Preference index, defined as the ratio of the number of times a mouse contacted any of the objects (training session) or the novel object (test session) to the total number of times the mouse contacted both objects, was used to measure object recognition memory.

#### 2.3.2 Object location test

The Object location test (OLT) was performed as for the Object recognition test (ORT) except that markers were placed outside of the box for recognition of the location, and the position of one of the objects was changed in test sessions. The preference index for the newly located object was calculated to measure object location memory.

### 2.4 Tissue collection

After behavioral testing, the mice were sacrificed by cervical dislocation and the brains were quickly removed from the skull and then frozen in powdered dry ice.

### 2.5 Nissl staining and cell count of hippocampal neurons

The brains were cut in 12 µm sections on the frontal plane using a cryostat (Leica Biosystems Nussloch GmbH), and the slices were fixed with 10% formalin solution. After three washes with distilled water, the sections were dipped in a working solution that was prepared by adding 0.1% acetic acid to cresyl purple solution (Muto Pure Chemicals Co., Ltd). The sections were permeabilized with Hemo‐D solution (Falma, Japan) and mounted with Entellan new (Merck). Stained sections were photographed with an EVOS FL Imaging System (Thermo Fisher Scientific), and the photographs were converted into grayscale images to standardize the intensity of the positive signals. Using Image J (National Institutes of Health), the threshold range was set to 0–112 for staining images. The pixel size of analyzed particles was set to 50‐infinity, and positive neurons were automatically counted. Results represent means ± standard error of the mean (*SEM*) (control group: *n* = 6; 1,000 mg/kg SD‐F‐treated group: *n* = 6).

### 2.6 RNA‐seq analysis

Total RNA was extracted from mouse hippocampal tissues using an RNeasy Mini Kit (Qiagen). The integrity of the extracted total RNA was analyzed using a Bioanalyzer (Agilent), and RNA‐seq libraries were constructed using the NEBNext Poly(A) mRNA Magnetic Isolation Module (NEB) and NEBNext Ultra II RNA Library Prep Kit for Illumina (NEB). The libraries (control vs. 1,000 mg/kg SD‐F, each *n* = 3) were sequenced on an Illumina Nextseq platform. Trimming, mapping, and read counts were performed using *Mus musculus* GRCm38 release‐92 as a reference sequence on CLC Genomics Workbench (CLC bio). Transcripts per million (TPMs) were calculated, and genes with low expression were eliminated (cutoff: TPM < 1). Genes with at least a 1.2‐fold change and *p*‐value < 0.05 were defined as differentially expressed genes (DEGs).

### 2.7 Quantitative real‐time PCR (qPCR)

Total RNA was used for cDNA synthesis using a PrimeScript RT reagent Kit with gDNA Eraser (Takara Bio Inc.) according to manufacturer's instructions. Quantitative real‐time PCR was carried out using a FastStart Essential DNA Green Master via LightCycler 96 system (Roche). *Gapdh* (*glyceraldehyde 3‐phosphate dehydrogenase*) was used as an internal control. Primer sequences are listed in Table 1. Relative gene expression data were analyzed using the 2^−ΔΔ^ *C*_T_ method.

**Table 1:** Primers used for quantitative real‐time PCR

Gene	Sequence (5’ to 3’)
*Gapdh*	Forward: AGGTCGGTGTGAACGGATTTG
	Reverse: TGTAGACCATGTAGTTGAGGTCA
*Egr1*	Forward: ACCTGACCACAGAGTCCTTTTC
	Reverse: AGCGGCCAGTATAGGTGATG
*Gadd45b*	Forward: TGAATGTGGACCCCGACAG
	Reverse: ATGCCTGATACCCGGACGAT
*Klk8*	Forward: GATCCTGGAAGGTCGAGAGTG
	Reverse: CTGCTCCGGCTGATCTCTG
*Nab2*	Forward: ATGAGCTGACCATCAACGAG
	Reverse: TGTCGGGACAGTGAGAAGAG
*Ngf*	Forward: GGGAGCGCATCGAGTGAC
	Reverse: CAAAACTCCACCATGCTGCC
*Ryr3*	Forward: GGCCGAGGTCTTCATTCTGT
	Reverse: GCATGGCTTTTGACATCTTGCT
*Vegfa*	Forward: CTTGTTCAGAGCGGAGAAAGC
	Reverse: ACATCTGCAAGTACGTTCGTT

### 2.8 Statistical analysis

In the behavioral tests, values are expressed as means ± *SEM*. In the RNA‐seq and qPCR analyses, values are expressed as means ± standard deviation (*SD*). Student's *t* test was used to determine the significance of differences between two groups. Differences with *p*‐values <.05 were considered significant.

## 3 RESULTS

### 3.1 Effect of new modified porcine placental extract (SD‐F) on body weight and food intake in aged mice

Male aged mice were fed a normal diet or SD‐F containing diet for 4 weeks. The weekly body weight and food intake during the 4‐week feeding period were monitored. The food intake and body weights among the groups were not different during the feeding period (Figure 1b,c). There were no adverse side effects in aged mice (data not shown).

### 3.2 Effect of SD‐F on object recognition and object location memory in aged mice

**Figure 2:**
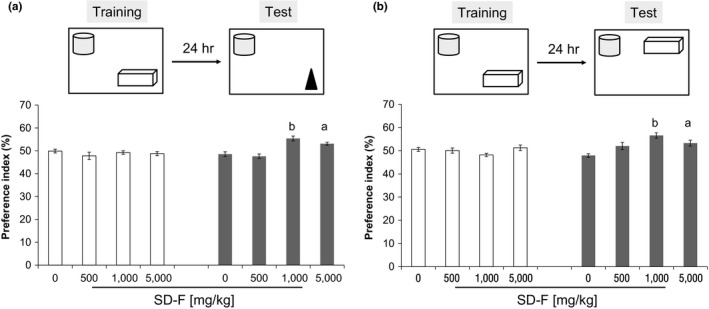
Effect of SD‐F in the object recognition test (ORT) and object location test (OLT) in aged mice. Four weeks after ingestion of SD‐F, behavioral tests were carried out. Result of the ORT (a). Preference index for the new object in the training and test sessions is shown. Result of the OLT. Preference index for the new object location in the training and test sessions is shown (b). Results represent means ± *SEM* (^a^ *p* < .05, ^b^ *p* < .001 vs. 0 mg/kg SD‐F‐treated group)

On the last day of feeding, the ORT was conducted. In the training session, all four groups showed approximately 50% in the preference index (Figure 2a). In the test session, the SD‐F feeding groups (1,000 and 5,000 mg/kg) showed significantly more frequent exploratory behavior in the presence of a novel object than that predicted by chance (50%). In contrast, the exploratory behavior in the presence of the novel object in the control group was close to chance (50%). An object location test was also carried out. As with the object recognition test (Yong and Tohda, 2018), the SD‐F feeding groups (1,000 and 5,000 mg/kg) showed significantly more frequent exploratory behavior in the presence of a newly located object than that predicted by chance (50%) (Figure 2b).

### 3.3 Effect of SD‐F on the number of neuronal cells in the hippocampal CA3 region in aged mice

**Figure 3:**
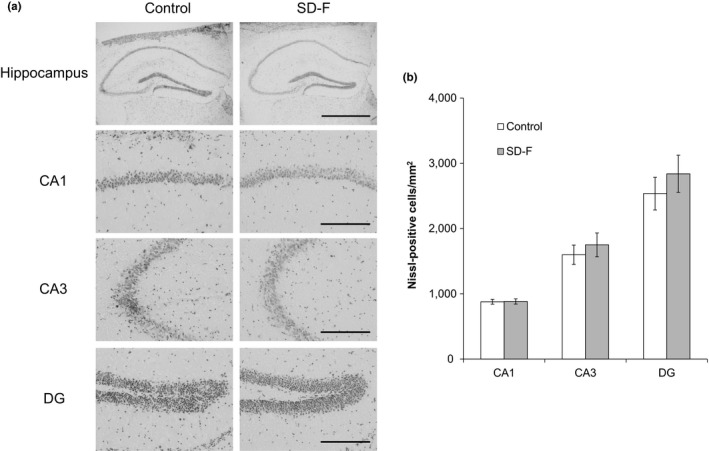
Representative photomicrographs displaying Nissl staining of hippocampal CA1, CA3, and dentate gyrus (DG) subfields of control and SD‐F‐treated aged mice. Nissl‐positive cells were visualized using cresyl violet staining. There was no significant difference between the groups. Results represent means ± *SEM* (control group: *n* = 6, 1,000 mg/kg SD‐F‐treated group: *n* = 6). Scale bars: Hippocampus = 1 mm; CA1, CA3, and DG = 0.2 mm (a). Cell numbers in the CA1, CA3, and DG subregions in aged mice treated with or without SD‐F (b)

There was no definite difference in Nissl‐positive cell layer thickness in the hippocampus between control and *SD*‐treated mice (Figure 3a). However, Nissl‐positive cells in SD‐F‐treated aged mice were integrated relative to those in control aged mice in the hippocampal cornu ammonis 3 (CA3) region (Figure 3a), and the number of Nissl‐positive cells in CA3 and the dentate gyrus (DG) was increased in SD‐F‐treated aged mice relative to control aged mice, though there was no significant difference between them (CA3; 1598 ± 361 vs. 1751 ± 407/mm^2^, *p* = .547 and DG; 2,535 ± 614 vs. 2,839 ± 635/mm^2^, *p* = .460, respectively) (Figure 3b).

### 3.4 RNA‐seq analysis of the hippocampus of aged mice treated with and without SD‐F

**Figure 4:**
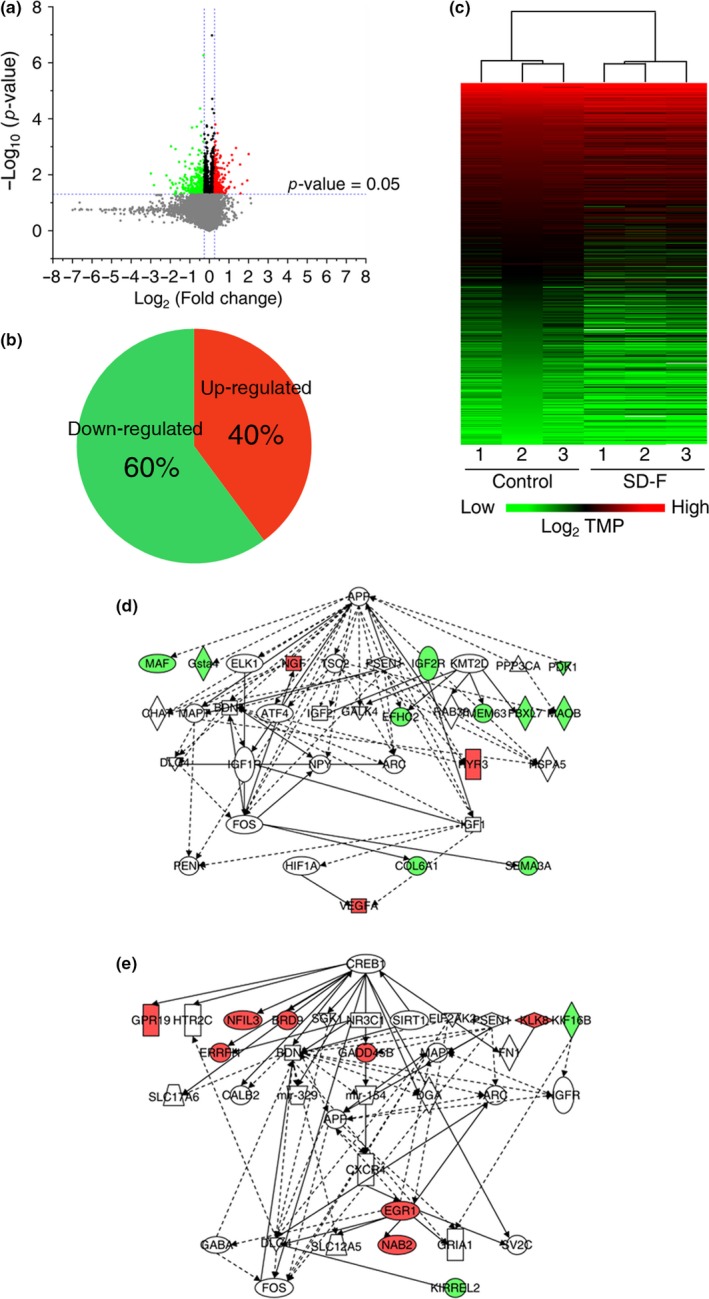
Genes involved in memory function in the hippocampus of aged mice treated with SD‐F. Volcano plot highlighting differentially expressed genes (a). Up‐regulated and down‐regulated genes are shown in green and red, respectively. Pie chart showing the percentage of genes up‐ and down‐regulated in control versus SD‐F (1,000 mg/kg) groups. Of the 542 differentially expressed genes, 216 were up‐regulated and 326 were down‐regulated (b). Heatmap of differentially expressed genes in control versus SD‐F (1,000 mg/kg) groups. High expression is shown by the red color spectrum, and low expression is shown by the green color spectrum (c). Two gene networks related to neurological function are shown. The networks are shown as pictures for genes and lines for biological relationship. Solid lines represent a direct interaction, and dotted lines represent indirect interactions between the genes. The shapes of genes indicate their molecular functions. Up‐regulated and down‐regulated genes are shown in red and green colors, respectively. Data are shown as means ± *SEM* (control group: *n* = 3, 1,000 mg/kg SD‐F‐treated group: *n* = 3) (d, e)

Total RNA was extracted from the hippocampi of control and SD‐F (1,000 mg/kg) groups after the behavioral tests, and mRNA‐sequencing (RNA‐seq) analysis was performed. Analysis of the data yielded 542 DEGs, of which 216 were up‐regulated and 326 were down‐regulated (Figure 4a). We observed a trend toward higher numbers of down‐regulated genes (60%) compared with up‐regulated genes (40%) (Figure 4b). A full list of the DEGs identified is included in Table S1. Clustering among the two groups (control and SD‐F) and their replicates using the DEG expression profiles showed that the two groups separated (Figure 4c). To search for pathways or genes responsible for the memory‐improving effects of SD‐F, DEGs were analyzed using IPA software. The gene network analysis returned several networks related to neurological functions, and the two networks where the scores were greater than 9 were further analyzed (Table 2 and Figure 4d,e). Eleven genes were up‐regulated in the two networks (shown in red), and seven genes (*early growth response protein 1* [*Egr1*], *growth arrest and DNA‐damage‐inducible, beta* [*Gadd45b*], *Klk8*, *Nab2*, *Ngf*, *Ryr3*, and *vascular endothelial growth factor a* [*Vegfa*]) were associated with memory function.

**Table 2:** Result of gene network analysis

ID	Molecules in network	Score	Focus molecules	Top diseases and functions
1	APP, ARC, ATF4, BDNF, CHAT, COL6A1, DLG4, EFHC2, ELK1, FBXL7, FOS, GALK1, Gsta4, HIF1A, HSPA5, IGF1, IGF2, IGF1R, IGF2R, KMT2D, MAF, MAOB, MAPT, NGF, NPY, PDK1, PENK, PPP3CA, PSEN1, RAB38, RYR3, SEMA3A, TMEM53, TSC2, VEGFA	13	13	Behavior, cell death and survival, neurological disease
2	APP, ARC, BDNF, BRD9, CALB2, CREB1, CXCR4, DLG4, EGR1, EIF2AK2, ERRFI1, FN1, FOS, GABA, GADD45B, GDA, GPR19, GRIA1, HTR2C, KIF16B, KIRREL2, KLK8, MAPT, mir−154, mir−329, NAB2, NFIL3, NGFR, NR3C1, PSEN1, SGK1, SIRT1, SLC12A5, SLC17A6, SV2C	9	10	Neurological disease, organismal injury and abnormalities, behavior
3	LRRK2, SNCA	1	1	Inflammatory disease, inflammatory response, neurological disease
4	CTTNBP2, STRN	1	1	Cell morphology, cellular assembly and organization, cellular development
5	EMX2, RELN	1	1	Embryonic development, nervous system development and function, organ development
6	mir−124, TRAF6	1	1	Neurological disease, organismal injury and abnormalities, psychological disorders

### 3.5 Validation of the expression of selected genes identified by RNA‐seq in the hippocampus of aged mice treated with and without SD‐F

**Figure 5:**
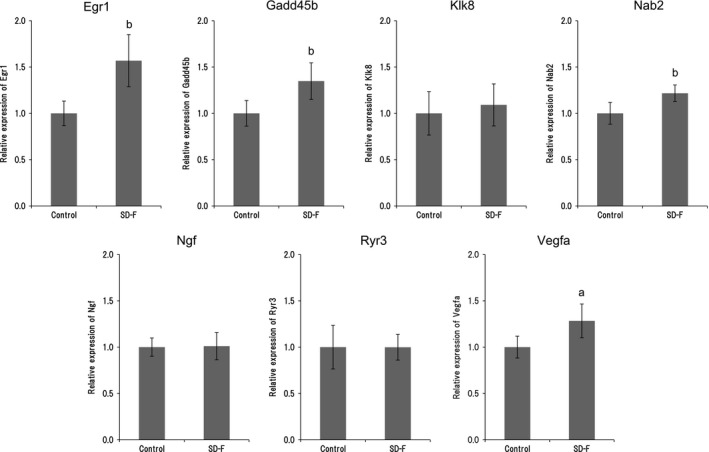
qPCR analysis. Relative expression of early growth response protein 1 (Egr1), growth arrest and DNA‐damage‐inducible, beta (Gadd45b), kallikrein‐8 (Klk8), NGFI‐A binding protein 2 (Nab2), nerve growth factor (Ngf), ryanodine receptor 3 (Ryr3), and vascular endothelial growth factor (Vegfa) in the hippocampus of aged mice treated with and without SD‐F (1,000 mg/kg). All data (mean ± *SD*, *n* = 9) were normalized to GAPDH expression. ^a^ *p* < .01, ^b^ *p* < .001 versus control group

Expression of the seven selected genes, that is, *Egr1*, *Gadd45b*, *Klk8*, *Nab2*, *Ngf*, *Ryr3*, and *Vegfa,* was confirmed by qPCR. The expression of *Egr1*, *Gadd45b*, *Nab2*, and *Vegfa* was significantly increased, and *Egr1* expression was highest out of all of these. The expression of *kallikrein‐8* (*Klk8*), *nerve growth factor* (*Ngf*), and *ryanodine receptor 3* (*Ryr3*) was unaltered (Figure 5).

## 4 DISCUSSION

In this study, we showed that SD‐F has a potentiating memory function in aged mice. It has been reported that age‐associated decrease in memory function in mice already occurred at 16 or 18 months of age, and it is prominent at 24 months of age (Peleg et al., 2010). Thus, it is suggested that SD‐F may be effective early in the onset of memory decline in age mice. On the other hand, since we have not examined the effect of SD‐F in memory function at young mice, it still remains unclear. However, it has demonstrated that porcine placental extract can improve memory impairment in 2‐month‐old mice (Takuma et al., 2012), and SD‐F may effect on memory function in young mice.

Apart from placental extracts, there have been many reports on dietary supplements, such as phosphatidylserine, plasmalogen, ferulic acid, curcumin, docosahexaenoic acid, and flavonoids, which have shown beneficial effects in aged humans and rodents (Barberger‐Gateau et al., 2007; Braverman & Moser., 2012; Hirayama et al., 2014; Mori, Koyama, Guillot‐Sestier, Tan, & Town, 2013; Rendeiro, Guerreiro, Williams, & Spencer, 2012; Yang et al., 2005). Despite an abundance of dietary supplements, they have often been shown to be ineffective in humans (Tan et al., 2015). It is suggested that this is owing to a lack of potency or targeting insufficiency. Given that one of the causes may be a of lack potency, to overcome the problem, it may be useful to induce synergistic effects by combined usage. In addition, since most dietary supplements are plant‐based, a combination of plant‐based and animal‐based products may be better than combining the same types of products. In fact, it has been reported that combined treatment with ferulic acid and octyl gallate or phenolics (‐)‐epigallocatechin‐3‐gallate improved cognition and neurodegeneration in an AD mouse model (Mori et al., 2017, 2019). Considering the above, it is possible that placental extract may be a new useful co‐agent.

Since placental extract contains nutritious ingredients, such as amino acids and minerals, in abundance (Hong, Lee, Hahn, Kim, & Lew, 2010; Jung et al., 2011), it had been expected to increase the body weight of mice. However, our present study showed that SD‐F‐treated aged mice did not have significant changes in either body weight or food intake relative to untreated controls. In addition, SD‐F did not induce adverse side effects in aged mice. Thus, this indicates that SD‐F may potentiate memory function in aged mice without affecting body weight or food intake, and therefore, it may be useful as a health supplement in humans. Further research on humans is essential to validate these findings.

The present study revealed that SD‐F improves memory, and that this occurs in a dose‐dependent manner. The efficacy of SD‐F was slightly decreased in aged mice treated with 5,000 mg/kg compared to 1,000 mg/kg, although adverse side effects were not observed. From these results, it is suggested that there may be threshold for the memory‐improving activity of SD‐F between these two doses. It is known that the hippocampal formation is essential for memory function and is linked to cognitive age‐associated memory impairment (Fanselow, 2010). Age‐associated memory decline has been shown in mice (Stilling et al., 2014). Moreover, the neuronal cells of the hippocampal CA3 region show progressive age‐associated decline (Bronzetti, Felici, Zaccheo, & Amenta, 1991; Brunson, Eghbal‐Ahmadi, Bender, Chen, & Baram, 2001; Hioki et al., 2009). It was expected that placental extract may reduce the loss of neuronal cells associated with age, as it is known to have cytoprotective effects (Kawakatsu, Urata, Goto, Ono, & Li, 2012; Kim et al., 2010; Liu et al., 2019). Porcine placental extract has previously been shown to suppress fear memory impairment and Nissl‐positive cell loss in the hippocampal CA3 region induced by restraint stress in ovariectomized mice (Takuma et al., 2012). However, the numbers of Nissl‐positive cells in the hippocampal CA3 and DG regions were increased in SD‐F‐treated aged mice relative to controls in this study, though this was not significant. We suggest that the memory‐enhancing effect of SD‐F may not be owing to effects on the number of neuronal cells in the hippocampal CA3 region in aged mice, but it may instead improve neuronal cell function.

To gain insight into the molecular basis of the memory‐improving effects of SD‐F, total RNA was extracted from the hippocampus of control and SD‐F (1,000 mg/kg)‐treated mice after the behavioral tests and RNA‐seq analysis was performed. Sequenced memory‐associated genes were selected from an IPA pathway database, which revealed a modest alteration in the expression of genes enriched for brain function processes, such as synaptic plasticity. In this study, we identified the memory‐associated genes. Four genes were significantly up‐regulated in aged mice treated with SD‐F relative to control, that is, *Erg1*, *Gadd45b*, *Nab2*, and *Vegfa*. These show different cellular localizations; *Erg1* and *Nab2* are localized in the cell nucleus, *Gadd45b* is localized to the cytoplasm, and *Vegfa* is secreted extracellularly (Gallo, Katche, Morici, Medina, & Weisstaub, 2018; Leach et al., 2012; Marballi & Gallitano, 2018; Mateo et al., 2006). *Erg1* is a member of the Erg family of immediate early gene transcription factors and plays an important role in long‐term memory formation and late‐phase long‐term potentiation (LTP) (Cole, Saffen, Baraban, & Worley, 1989; Worley et al., 1993). *Nab2* is an activity‐dependent immediate early gene that functions as a transcriptional coregulatory protein by binding to a specific recognition domain on EGR1. Interestingly, EGR1 protein binds to the *Nab2* promoter region and then induces its expression in neuroectodermal cells (Kumbrink, Kirsch, & Johnson, 2010). In addition, once bound to the EGR1, Nab2 feedback suppresses its own expression (Kumbrink et al., 2010; Svaren et al., 1996). Thus, Nab2 protein plays a role in both co‐activation and corepression. In this study, it was observed that the expression of both *Egr1* and *Nab2* is increased in SD‐F‐treated aged mice relative to control mice. Since the expression level of *Egr1* and *Nab2* genes differed by 1.3‐fold, the possibility of negative feedback by Nab2 cannot be denied. As studies on the role of *Nab2* in the brain and in memory are limited, the function of *Nab2* in hippocampal synaptic plasticity or behavior remains to be clarified. Despite this, memory‐enhancing effects were shown in SD‐F‐treated aged mice relative to control mice in this study; thus, SD‐F may be a useful tool to investigate the role of *Nab2* in the brain. *Gadd45b* is essential for active demethylation of the promoter regions of select genes (Ma et al., 2009; Wu & Sun, 2009) and modulates memory and synaptic plasticity (Leach et al., 2012; Sultan, Wang, Tront, Liebermann, & David Sweatt, 2012). *Gadd45b* mapped onto the same gene network as *Egr1* and *Nab2*. The increased expression of *Gadd45b* coincided with that of *Egr1* and *Nab2* in SD‐F‐treated aged mice, though there was no direct or indirect relationship among them in this network (Figure 4e). The link between them is that they may be regulated by *PSEN1*, which is located upstream of these factors. *PSEN1* codes for the presenilin‐1 protein, which functions as a peptidase. As *PSEN1* was not identified by the RNA‐seq analysis in this study, there may not be a direct relationship between the memory‐enhancing effects of SD‐F and *PSEN1* expression. This suggests that the upregulation of *Gadd45b* by SD‐F may be independent of the regulation of *PSEN1*. VEGF is a major regulator of pre‐existing (angiogenesis) and de novo vessels (vasculogenesis). It has been reported that VEGF plays a role in memory formation and learning, which are mediated by angiogenesis and neurogenesis in the rat hippocampus (Cao et al., 2004; Sun, Sha, Zhou, & Yang, 2010). In this study, the network in which *Vegfa* was involved differed from that of *Egr1*, *Nab2,* and *PSEN1*. The genes located upstream of *Vegfa* in this network are *HIF1A* and *GF1*. The former has a direct relationship with *Vegfa*, while the latter has an indirect relationship (Figure 4d). As *GF1* is also located upstream of *HIF1A*, the expression of *Vegfa* could be regulated by *GF1*. Since the expression of *GF1* was not altered in SD‐F‐treated aged mice relative to control mice, SD‐F may act directly on the expression of *Vegfa,* without acting on *GF1,* in aged mice. The expression of *HIF1A* is similar to *GF1*, and nuclear translocation of HIF1A for *Vegfa* expression is even more important than for its own expression; thus, we cannot deny the possibility that SD‐F influences it.

## 5 CONCLUSION

Our results showed that SD‐F could ameliorate the age‐associated decrease in memory function in mice. The effect of SD‐F on the hippocampus of aged mice may be mediated via the upregulation of the four genes: Egr1, Nab2, Gadd45b, and Vegfa. Further clinical trials on the efficacy, safety, and mode of action of SD‐F in humans are recommended.

## CONFLICT OF INTEREST

Taiichi Kaku is a stockholder of Japan Bio Products Co., Ltd. Akihiro Yamauchi, Takahiro Tone, Koji Sugimoto, Eiichi Hirano, and Hong Seok Lim are employees of Japan Bio Products Co., Ltd. Chihiro Tohda, Takayuki Shindo, Koji Tamada, and Yoichi Mizukami declare no conflict of interest.

## ETHICAL APPROVAL

This study has not any potential sources of conflict of interest. This study was conducted in accordance with the Declaration of Helsinki, and all animals were housed and cared at the Division of Laboratory Animal Research, Department of Life Science, Research Center for Human and Environmental Sciences, Shinshu University (Matsumoto, Japan) in accordance with protocols approved by the Ethics Committee of the International Animal Care and Use Committee and NIH guidelines.
